# Weight-based dosing of surgical antibiotic prophylaxis in patients with obesity: meta-analysis

**DOI:** 10.1093/bjsopen/zrag015

**Published:** 2026-04-17

**Authors:** Hiske Huisman, Karlijn Huinink, Nathan Bontekoning, Stijn W de Jonge, Gerjon Hannink, Paulina Salminen, Marja A Boermeester

**Affiliations:** Department of Surgery, Amsterdam UMC Location University of Amsterdam, Amsterdam, the Netherlands; Amsterdam Gastroenterology Endocrinology and Metabolism, Amsterdam, the Netherlands; Department of Surgery, Amsterdam UMC Location University of Amsterdam, Amsterdam, the Netherlands; Amsterdam Gastroenterology Endocrinology and Metabolism, Amsterdam, the Netherlands; Department of Surgery, Amsterdam UMC Location University of Amsterdam, Amsterdam, the Netherlands; Amsterdam Gastroenterology Endocrinology and Metabolism, Amsterdam, the Netherlands; Department of Surgery, Amsterdam UMC Location University of Amsterdam, Amsterdam, the Netherlands; Amsterdam Gastroenterology Endocrinology and Metabolism, Amsterdam, the Netherlands; Department of Medical Imaging, Radboud University Medical Center, Nijmegen, the Netherlands; Division of Digestive Surgery and Urology, Turku University Hospital, Turku, Finland; Department of Surgery, University of Turku, Turku, Finland; Department of Surgery, Amsterdam UMC Location University of Amsterdam, Amsterdam, the Netherlands; Amsterdam Gastroenterology Endocrinology and Metabolism, Amsterdam, the Netherlands

## Abstract

**Background:**

The use of preoperative surgical antibiotic prophylaxis is effective in preventing surgical site infection. However, obesity, a major risk factor for surgical site infection, affects the pharmacokinetics and effectiveness of surgical antibiotic prophylaxis. Evidence for weight-based surgical antibiotic prophylaxis in patients with obesity is inconsistent.

**Methods:**

MEDLINE (PubMed), Embase, CENTRAL, and CINAHL were searched up to 21 October 2025 for eligible studies on weight-based surgical antibiotic prophylaxis and surgical site infection. This systematic review and random-effects meta-analysis compared weight-based dosing of surgical antibiotic prophylaxis with standard surgical antibiotic prophylaxis, in terms of surgical site infection rates in patients with obesity. The certainty of evidence was evaluated using the Revised Cochrane risk-of-bias tool for randomized trials, the Risk Of Bias in Non-randomized Studies—of Interventions tool for observational studies, and Grading of Recommendations Assessment, Development and Evaluation (GRADE).

**Results:**

Of 2782 potentially relevant articles, 33 studies were eligible (3 randomized clinical trials, 30 observational). A total of 99 211 patients were included, of whom 2362 (2.4%) developed a surgical site infection. Risk of bias varied from ‘low’ to ‘some concerns’ in randomized trials, and ‘some concerns’ to ‘serious’ in observational studies. Meta-analysis of 3 randomized trials with only 1 surgical site infection among 103 patients (1.0%) showed no significant reduction in surgical site infection rates in patients receiving weight-based dosing of cefazolin *versus* standard dosing (risk difference 2.02 (95% confidence interval −3.15 to 7.19)%). Meta-analysis of 6 observational studies with 45 554 patients and 610 surgical site infections (1.3%) showed significantly reduced surgical site infection rates in patients receiving weight-based dosing of cefazolin *versus* standard dosing (risk difference −1.93 (−2.84 to −1.02)%), with most studies focusing on orthopaedic surgery. GRADE assessments showed very low certainty of evidence.

**Conclusion:**

Based on observational data, the use of weight-based dosing of surgical antibiotic prophylaxis may reduce the risk of surgical site infection in patients with obesity compared with standard dosing, but the existing evidence is very uncertain.

## Teaser

This study comprised a systematic review and meta-analysis of all available evidence for the effect of weight-based dosing of surgical antibiotic prophylaxis on the incidence of surgical site infection in patients with overweight and obesity. Based on data from observational studies, the findings suggest that the use of weight-based dosing of surgical antibiotic prophylaxis may reduce the risk of surgical site infection in patients with obesity compared with standard dosing of surgical antibiotic prophylaxis, but the existing evidence is very uncertain.

## Introduction

Obesity is a progressive multifactorial chronic disease and its prevalence is rising^1^. With approximately 30% of the global population being overweight or obese, the condition is considered one of the major global health threats, and the obesity pandemic poses a severe healthcare burden both on patients and society^1–3^. Increasing numbers of operations are being performed on this population^4^. In addition to the general healthcare burden on patients and society, obesity is also associated with an increased risk of developing surgical site infections (SSIs)^5,6^. In turn, SSIs are associated with higher healthcare costs, readmissions, prolonged hospital stay, and increased risk of death^7,8^.

Preoperative surgical antibiotic prophylaxis (SAP), when indicated, is arguably the most important measure to help prevent SSIs^9–11^. The most common species isolated from SSIs after clean procedures include *Staphylococcus aureus* and coagulase-negative staphylococci. In clean-contaminated procedures, including abdominal surgery, the predominant species include Gram-negative bacteria and enterococci^6,9^. Owing to their broad spectrum of activity, being well tolerated and with a low incidence of allergy, first-generation cephalosporins, such as cefazolin, are the SAP choice for most procedures^12^. However, the physiological changes resulting from obesity lead to altered tissue distribution, with potentially insufficient tissue concentrations for the antibiotics to be effective^13,14^. Therefore, standard doses of SAP may not be sufficient to achieve the minimum inhibitory concentration (MIC) in serum and tissue concentrations for the most common pathogens to prevent SSI^9^. This may in part explain the increased risk of SSI observed in patients with obesity^15^.

Adjusting antibiotic dosage according to bodyweight may compensate for these physiological changes and has been recommended for various antibiotics^9^. A tailored approach that considers patient- and procedure-specific characteristics and features has already been suggested in other areas, such as extended pharmacological thromboprophylaxis after major surgery^16^. Similarly, individualized dosing of SAP may be warranted in patients with obesity.

However, evidence on weight-based dosing of SAP is conflicting. A recent review^17^ of pharmacokinetic studies suggested that there is no evidence to increase one-time preoperative dosing of cefazolin beyond 2-g in patients with obesity undergoing surgery lasting up to 4 h. This is in contrast with existing guidelines, in which a 3-g dose is recommended for patients weighing more than 120 kg^9^. A review^18^ published in 2014 stated that the use of cefazolin is recommended in metabolic bariatric surgery, but was inconclusive about dosing strategies. Moreover, an up-to-date quantitative analysis is needed not only for metabolic bariatric surgery, but for all types of surgery and other types of SAP. This systematic review and meta-analysis aimed to summarize the current evidence on the effect of weight-based dosing of SAP in patients with obesity for the prevention of SSI in all types of surgery.

## Methods

### Search strategy and selection criteria

This study is reported in accordance with the PRISMA statement^19^. The study protocol is available in the PROSPERO database (CRD42024591244).

All published clinical studies comparing different dosing regimens in patients with obesity, or comparing different weight categories under the same dosing regimens in patients with obesity, were eligible for inclusion. There was no restriction on language. Studies before 2000 were excluded as they were unlikely to adhere to the current standards of perioperative care as suggested by Mangram *et al.*^20^. MEDLINE (PubMed), Excerpta Medica Database (Embase), Cochrane Central Register of Controlled Trials (CENTRAL), and Cumulative Index to Nursing and Allied Health Literature (CINAHL) were searched up to 21 October 2025. The detailed search strategy can be found in the *supplementary methods*.

### Study selection

Preliminary evaluation of articles was undertaken by two independent reviewers (H.H. and K.H.). Titles and abstracts of retrieved records were screened for relevance. Duplicates, conference abstracts, meta-analyses, and *in vitro*/*in vivo* studies were not included. Disagreements regarding eligibility of references were resolved through discussion or, when necessary, after consultation with a senior author (N.B. or M.A.B). Next, full texts of references that potentially met the inclusion criteria were obtained and evaluated. All studies that investigated the incidence of SSI as a primary or secondary outcome parameter for different doses of SAP in patients with overweight or obesity, or studies comparing different weight categories under the same dosing regimens, were considered eligible. Overweight or obesity was defined at the author’s discretion. As the literature suggests that a 1-g dose of cefazolin may be inadequate for patients with obesity exceeding the MIC^9^, only studies using ≥ 2-g for patients with obesity in the intervention group were included. No clear data on specific dosing is available for other antibiotics, so no restrictions on inclusion were made. Studies that explored only plasma and tissue concentrations were excluded. Procedures from all surgical fields and all types of antibiotic regimen were included.

### Quality assessment

Both reviewers independently assessed all articles for risk of bias. Randomized clinical trials (RCTs) were appraised with the Revised Cochrane risk-of-bias tool for randomized trials^21^ and observational studies with the Risk Of Bias in Non-randomized Studies—of Interventions^22^ assessment tool. Any disagreement between reviewers was resolved by discussion or consultation with a senior author (N.B. or M.A.B.).

### Data extraction

Data necessary for analyses were extracted from the articles by both reviewers. Extracted data included: study design, number of patients, specialty, wound classification based on Centers for Disease Control and Prevention (CDC) level^10^, weight categories (including number of patients), type of SAP, dosing of SAP, SSI counts with incidence, statistics, and follow-up time. If there were missing data on SSI incidence (per weight category or dosing group), authors were contacted to obtain the necessary information, and one further request was sent if required. Discrepancies in data entry were resolved by discussion.

### Outcome measures

The primary outcome was the incidence of all types of SSI (superficial, deep, and organ space).

### Statistical analysis

Appropriate data were summarized in a meta-analysis. Exclusion criteria for the meta-analysis included use of second-generation cephalosporins, non-comparable study groups (for example large differences in body mass index (BMI) across different dosing comparison groups), or failure to specify which study arm the SSI occurred in. Unadjusted crude data were used. A random-effects meta-analysis was conducted using the inverse-variance method. When studies contained no events in one or both groups, a continuity correction of 0.5 was applied. Between-study variance was quantified using the τ^2^ statistic, estimated using the restricted maximum likelihood estimator with Hartung–Knapp adjustment. Heterogeneity was assessed by visual inspection of forest plots, use of the *I*^2^ statistic (an *I*^2^ value of < 25% was considered to indicate low, between 25 and 50% moderate, and > 50% high heterogeneity) and its connected χ^2^ test, and 95% prediction intervals (PIs) were calculated to indicate the expected range of true effects. Pooled effects were displayed as risk differences (RDs) with 95% confidence intervals instead of odds ratios (ORs) to facilitate clinical interpretability. *P* < 0.050 was considered statistically significant.

To assess the robustness of RD results, an arcsine-difference model was implemented as an alternative approach in a sensitivity analysis^23^. In addition, a random-effects meta-analysis for the use of cefazolin only, without the additional use of metronidazole, was undertaken as sensitivity analysis to assess potentially relevant differences. Finally, to enable readers to evaluate both absolute and relative perspectives of the effect, while preserving the clinical interpretability of the primary results, an additional analysis was undertaken using the OR in a random-effects model.

R version 4.3.2 (R Foundation for Statistical Computing, Vienna, Austria) was used to perform the statistical analyses.

### Assessment of certainty of evidence

The certainty of evidence for the meta-analyses was assessed by means of the Grading of Recommendations, Assessment, Development and Evaluations (GRADE), using the GRADEpro guideline development tool^57^.

## Results

### Systematic review

**Fig. 1:**
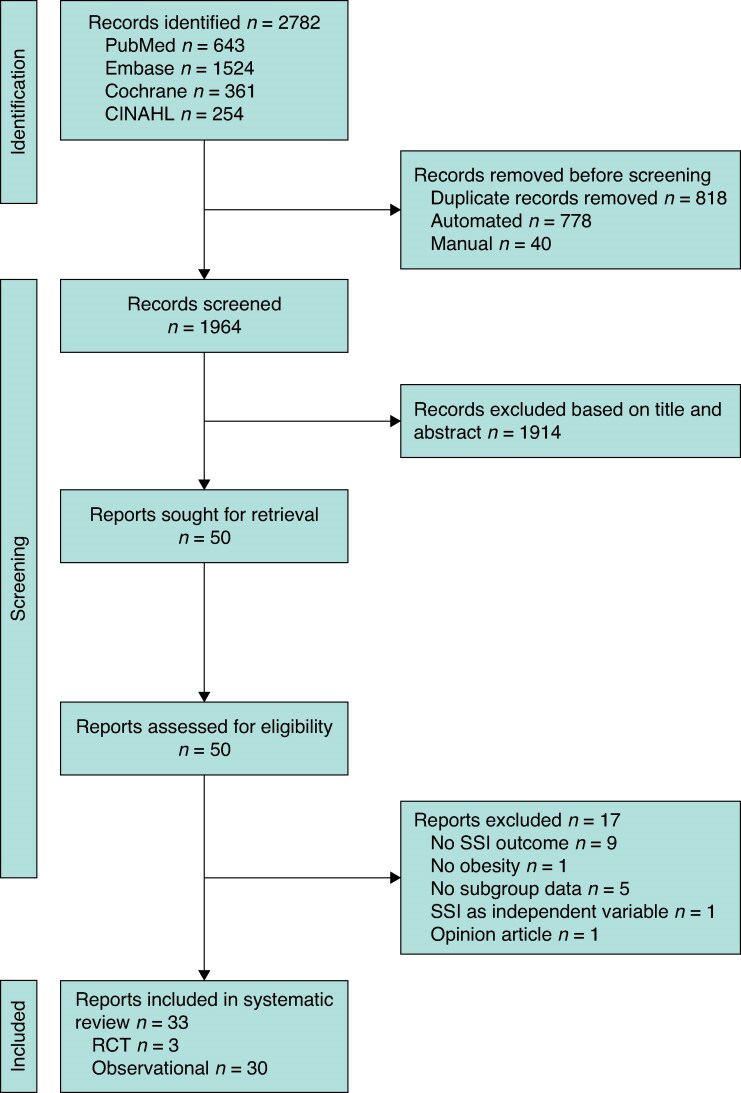
PRISMA flow chart showing selection of articles for review SSI, surgical site infection; RCT, randomized clinical trial.

The search identified 2782 potentially relevant records. After title and abstract screening, 50 full-text articles were assessed, of which 33 studies were included in the systematic review. Screening and study selection are summarized in *Fig. 1* and reasons for exclusion after full-text screening are shown in *Table S1*.

### Study characteristics

Study characteristics are summarized in *Table 1*; an expanded version of this table is available in the Supplementary material (*Table S2*). In total, three RCTs^24–26^ were included. The included observational studies comprised 20 comparative studies (7 prospective^32--35,39,40,45^, 13 retrospective^27–31,36–38,41–44,46^) and 10 single-arm studies (7 prospective^47–52,54^ and 3 retrospective^53,55,56^). A total of 99 211 patients were included across all 33 studies, of whom 2362 (2.4%) developed SSIs. The studies were published between 2004 and 2025, and they encompassed a range of surgical procedures, including bariatric surgery, colorectal surgery, obstetrics, gynaecology, orthopaedics, and trauma surgery. Sample sizes varied widely, from as few as 10 to as many as 37 640 patients. All studies described the effect of use of weight-based SAP on SSI incidence. SSI incidence was either reported as a primary or secondary outcome. All RCTs reported SSI incidence as a secondary outcome. Only 15 of 33 studies followed the CDC definition for SSI diagnosis^10^.

**Table 1:** Characteristics of included studies

Reference	*n*	Specialty	Weight categories*	Type of SAP	Dose*	Primary outcome	SSI	Risk of bias
**RCTs**								
Maggio *et al*.^24^†	57	Obstetrics	BMI ≥ 30 kg/m^2^	Cefazolin	I: 3 g (29) II: 2 g (28)	ATC	I: 1 of 29 (3.4%) II: none	Some concerns
Stitely *et al.*^25^†	20	Obstetrics	BMI ≥ 35 kg/m^2^	Cefazolin	I: 4 g (9) II: 2 g (11)	PC	None	Low
Young *et al*.^26^†	26	Obstetrics	BMI ≥ 30 kg/m^2^	Cefazolin	I: 3 g (13) II: 2 g (13)	PC	None	Low
**Observational comparative studies**								
Ahmadzia *et al*.^27^	335	Obstetrics	≥ 290 pounds	Cefazolin	I: 3 g (160) II: 2 g (175)	SSI	I: 21 of 160 (13.1%) II: 23 of 175 (13.1%)	Serious
Banoub *et al*.^28^	175	General, gynaecology	≥ 120 kg	Cefotetan/cefoxitin	I: 3 g (35) II: 2 g (140)	SSI	I: 8 of 35 (22.9%) II: 29 of 140 (20.7%)	Some concerns
Catanzano *et al*.^29^	216	Orthopaedics	n.s.	Vancomycin	Underdosed: < 1 g (149) Appropriate: 1 g (45) Overdosed: > 1 g (22)	PC	Underdosed: 6 of 149 (4.0%) Appropriate: none Overdosed: none	n.s.
Collins *et al*.^30^†	581	Colorectal surgery	≥ 120 kg	Cefazolin/metronidazole	I: 3 g + 500 mg (367) II: 2 g + 500 mg (214)	SSI	I: 23 of 367 (6.3%) II: 16 of 214 (7.5%)	Serious
Doi *et al*.^31^†	121	Orthopaedics	≥ 80 kg	Cefazolin	I: 2 g (55) II: 1 g (66)	SSI	I: 0 of 55 (0%) II: 2 of 66 (3.0%)	Serious
Ferraz *et al*.^32^	363	Bariatric surgery	BMI ≥ 40 kg/m^2^	I: Ampicillin/sulbactam II: Ceftriaxone	I: 3 g (83) II: 1 g (280)	SSI	I: 5 of 83 (6.0%) II: 19 of 280 (6.8%)	Serious
Ferraz *et al*.^33^	896	Bariatric surgery	BMI ≥ 40 kg/m^2^	I: Ampicillin/sulbactam II: Ertapenem III: Cefazolin	I: 2 g/1 g (194) II: 1 g (303) III: 2 g (399)	SSI	I: 8 of 194 (4.1%) II: 6 of 303 (2.0%) III: 6 of 399 (1.5%)	Serious
Ferrer Pomares *et al*.^34^	84	Orthopaedics	BMI ≥ 30 kg/m^2^	A: Cefazolin (every 8 h for 24 h) B: Cefazolin + amikacin (every 8 h for 24 h) C: Cefazolin + amikacin (every 8 h for 74 h)	A: 2 g (30) B: 2 g + 500 mg (30) C: 2 g + 500 mg (24)	SSI	A: 8 of 20 (40.0%) B: 4 of 30 (13.3%) C: 2 of 24 (8.3%)	Serious
Fouks *et al*.^35^	42	Obstetrics	I: ≥ 80 kg (21) II: < 80 kg (21)	Cefazolin	I: 2 g (21) II: 1 g (21)	PL	None	n.s.
Hasler *et al*.^36^	7106	Orthopaedics	≥ 80kg	Cefuroxime	I: 3 g (3096) II: 1.5 g (4010)	SSI	I: 8 of 3096 (0.3%) II: 16 of 4010 (0.4%)	Serious
Hopkins *et al*.^37^	1273	Obstetrics	BMI ≥ 40 kg/m^2^	I: Cefazolin II: Cefazolin + azithromycin	I: 3 g (303) II: 3 g + 500 mg (970)	SSI	I: 31 of 303 (10.2%) II: 65 of 970 (6.7%)	Some concerns
Karamian *et al*.^38^†	2643	Orthopaedics	A: < 60 kg (258) B: 60–120 kg (2194) C: ≥ 120 kg (191)	Cefazolin	Recommended dose: 1 g if < 60 kg, 2 g if 60–120 kg, and 3 g if > 120 kg (1824) Underdosed: (819)	SSI	Recommended dose: 47 of 1824 (2.6%) Underdosed: 48 of 819 (5.9%)	Serious
Morris *et al*.^39^†	38 289	Orthopaedics	A: < 80 kg (15 114) B: 80–120 kg (21 164) C: ≥ 120 kg (2011)	Cefazolin	Recommended dose: 1 g if < 80 kg, 2 g if 80–120 kg, and 3 g if > 120 kg (36 183) Underdosed: (2106)	SSI	Recommended dose: 355 of 36 183 (1.0%) Underdosed: 53 of 2106 (2.5%)	Serious
Okoro *et al*.^40^†	768	Orthopaedics	n.s.	Cefazolin	New regimen: 2 g if < 120 kg, and 3 g if ≥ 120 kg (458) Old regimen: 2 g (310)	SSI	New regimen: 9 of 458 (2.0%) Old regimen: 9 of 310 (2.9%)	Serious
Peppard *et al*.^41^	436	Neurosurgery, orthopaedics, general or emergency trauma	≥ 100 kg	Cefazolin	I: 3 g (284) II: 2 g (152)	SSI	I: 21 of 284 (7.4%) II: 11 of 152 (7.2%)	Some concerns
Perez *et al*.^42^	816	Obstetrics	BMI ≥ 30 kg/m^2^	I: Cefazolin II: Cefazolin + azithromycin	I: 2 g if > 80 kg, 3 g if > 160 kg (525) II: As above + 1 g azithromycin (291)	SSI	I: 25 of 525 (4.8%) II: 6 of 291 (2.1%)	Some concerns
Salm *et al*.^43^	2161	Visceral, vascular, orthopaedics or trauma‡	≥ 80 kg	Cefuroxime + metronidazole	Double dose: 3 g + 1 g (1615) Single dose: 1.5 g + 500 mg (546)	SSI	Double-dose: 73 of 1615 (4.5%) Single-dose: 95 of 546 (17.4%)	Some concerns
Scheck *et al*.^44^	986	Obstetrics	BMI ≥ 30 kg/m^2^	Cefazolin	I: 3 g (731) II: 2 g (255)	SSI	n.s.	Serious
Sommerstein *et al*.^45^	37 640	Various§	≥ 80 kg	Cefuroxime	I: 3 g (13 246) II: 1.5 g (24 394)	SSI	I: 462 of 13 246 (3.5%) II: 747 of 24 394 (3.1%)	Some concerns
Wu *et al*.^46^†	3152	Orthopaedics	n.s.	Cefazolin	Optimal dose: 2 g if ≥ 80 kg and 1 g if < 80 kg (2846) Non-optimal dose: (306)	SSI	Optimal dose: 36 of 2846 (1.3%) Non-optimal dose: 12 of 306 (4.0%)	Serious
**Observational single-arm studies**								
Belveyre *et al*.^47^	183	Bariatric surgery	BMI ≥ 35 kg/m^2^	Cefoxitin	4 g (183)	PC	2 of 183 (1.1%)¶	Some concerns
Chen *et al*.^48^	37	Bariatric surgery	BMI ≥ 35 kg/m^2^	Cefazolin	2 g (37)	SC	None	n.s.
Cinotti *et al*.^49^	116	Bariatric surgery	A: BMI 40–50 kg/m^2^ (79) B: BMI 50.1–65 kg/m^2^ (37)	Cefazolin	4 g (116)	ATC	None	Serious
Edmiston *et al*.^50^	38	Bariatric surgery	A: BMI 40–49 kg/m^2^ (17) B: BMI 50–59 kg/m^2^ (11) C: BMI ≥ 60 (10)	Cefazolin	2 g (38)	SC	A: 3 of 17 (17.6%) B: 1 of 11 (9.1%) C: 3 of 10 (30%)	Serious
Hites *et al*.^51^	63	Bariatric surgery	A: BMI < 35 kg/m^2^ (20) B: BMI ≥ 35 kg/m^2^ (43)	Cefazolin	2 g (63)	SC	A: 0 of 20 B: 1 of 43 (2.3%)	Serious
Hollis *et al*.^52^	10	Cardiology	≥ 120 kg (1)	Cefazolin	2 g (10)	SC	None	n.s.
Hussain *et al*.^53^	304	General, gynaecology and obstetrics, orthopaedics	Non-obese: < 120 kg (152) Obese: ≥ 120 kg (152)	Cefazolin	2 g (304)	SSI	Non-obese: 7 of 152 (4.6%) Obese: 13 of 152 (8.6%)	Serious
Moine *et al*.^54^	30	Bariatric surgery	A: BMI ≥ 40 kg/m^2^ (25) B: BMI < 40 kg/m^2^ (5)	Cefoxitin	40 mg/kg TBW (30)	SC	None	n.s.
Rodríguez de Castro *et al*.^55^	49	Trauma (orthopaedic and non-implant trauma surgery)	Non-obese: < 100 kg or BMI < 30 kg/m^2^ (26) Obese: ≥ 100 kg or BMI ≥ 30 kg/m^2^ (23)	Cefazolin	2 g (49)	SSI	Non-obese: 2 of 26 (7.7%) Obese: 2 of 23 (8.7%)	Serious
Unger and Stein^56^	195	Various§	Non-obese: BMI < 30 kg/m^2^ (96) Obese: BMI ≥ 30 kg/m^2^ (99)	Cefazolin	2 g (195)	SSI	Non-obese: 7 of 96 (7.3%) Obese: 5 of 99 (5.1%)	Serious

Values are *n* (%) unless stated otherwise; *values in parentheses are number of patients. †Included in meta-analysis. ‡Not clear whether emergency trauma surgery was included. §Potentially including: bariatric, cardiology, general, gynaecology, neurosurgery, orthopaedics, plastics, podiatry, trauma or vascular. ¶One surgical site infection (SSI) occurred in 9 patients receiving 2 g cefoxitin but these patients were excluded from analysis. SAP, surgical antibiotic prophylaxis; RCT, randomized clinical trial; BMI, body mass index; ATC, adipose tissue concentration; PC, plasma concentration; n.s., not stated; h, hours; PL, plasma level; TC, tissue concentration; SC, serum concentration; TBW, total bodyweight.

Weight inclusion criteria consisted of BMI or a more pragmatic strategy using bodyweight in kilograms and pounds; cut-off values varied between studies. All but seven studies^35,38,39,51,53,55,56^ specifically included patients with obesity (BMI ≥ 30 kg/m^2^ or weight ≥ 80 kg). In five studies^31,35,36,43,45^, the weight category also included patients with overweight, with a cut-off value of ≥ 80 kg most frequently used. In three studies^29,40,46^, numbers in different bodyweight categories and BMI were not reported. Primary outcomes were described as SSI incidence (21 studies)^27,28,30–34,36–46,53,55,56^, adipose tissue concentrations (2)^24,49^, or plasma or serum concentrations (10)^25,26,29,35,47,48,50–52,54^.

Nine studies^24–26,30,31,38–40,46^ that compared weight-based dosing of cefazolin with standard dosing were eligible for meta-analysis. Of these, all of the RCTs focused on obstetric procedures, whereas the observational studies focused on orthopaedic procedures except for one study^30^ in colorectal surgery. In the RCTs, the definition used for obesity was a BMI of ≥ 30 kg/m^2^ in two studies^24,26^ and ≥ 35 kg/m^2^ in one^25^, for which an adjusted dose of 3-g^24,26^ or 4-g^25^ was used in the intervention group, compared with 2-g in the control group. Four of the observational studies^30,31,39,46^ administered an adjusted dose of 2-g for a bodyweight ≥ 80 kg and 3-g for a bodyweight ≥ 120 kg. One study^38^ used a lower threshold, administering 2-g for patients weighing ≥ 60 kg and 3-g for those weighing ≥ 120 kg. One study^40^ used a cut-off value of ≥ 120 kg to administer 3-g, compared with 2-g for patients with a bodyweight of < 120 kg. Finally, one study^30^ in colorectal surgery administered metronidazole in addition to cefazolin as prophylaxis.

### Characteristics of antibiotic prophylaxis

An overview of types of SAP used and dosing is presented in *Table 1*, with further details in *Table S2*. The most frequently used type of SAP was cefazolin (22 studies). Other studies included cefotetan/cefoxitin^28,47,54^, cefuroxime^36,43,45^, metronidazole alongside cefazolin^30^, metronidazole alongside cefuroxime^43^, vancomycin^29^, ceftriaxone^32^, ertapenem^33^, ampicillin/sulbactam^32,33^, azithromycin alongside cefazolin^37,42^, or amikacin alongside cefazolin^34^. All comparative studies (RCT and observational) assessed different dosing regimens with respect to SSI incidence. Dosing groups were either categorized as specific gram amounts per intervention group or as standard SAP compared with weight-based regimens (‘recommended’, ‘optimal’, ‘appropriate’, ‘new regimen’). In the single-arm observational studies^47–56^, a uniform prophylactic dose was used without a comparison. These studies evaluated SSI incidence across different weight categories.

### Meta-analysis of studies evaluating weight-based dosing of cefazolin in patients with obesity

Nine studies^24–26,30,31,38–40,46^ comparing weight-based dosing of SAP *versus* standard dosing of cefazolin were included in the meta-analysis, comprising three RCTs and six observational two-arm studies. Together, these studies enrolled 45 657 patients with an overall low SSI incidence of 1.3%.

**Fig. 2:**
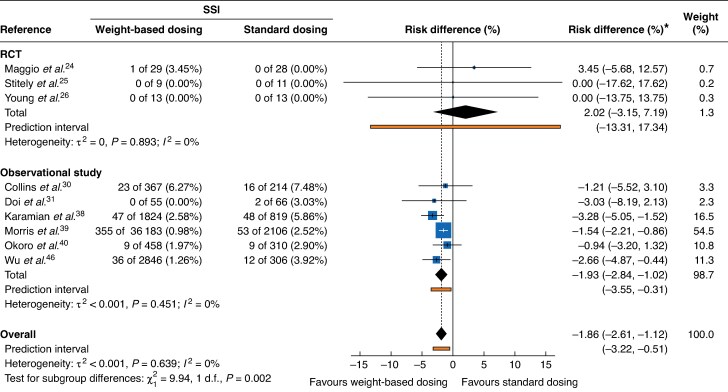
Meta-analysis of RCTs and observational studies using weight-based dosing of surgical antibiotic prophylaxis *versus* standard dosing in elective surgery procedures Values are *n* (%) unless otherwise stated. Risk differences are shown with 95% confidence intervals. Prediction intervals, represented by horizontal bars, illustrate the expected range of true effects. RCT, randomized clinical trial; SSI, surgical site infection.

Only one SSI was found among the three RCTs. Meta-analysis of these studies—including 103 patients and 1 SSI (SSI rate 1.0%)—showed no statistically significant difference in SSIs between weight-based dosing of SAP *versus* standard dosing (RD 2.02 (95% confidence interval (c.i.) −3.15 to 7.19)%; 95% PI −13.31 to 17.34%) (*Fig. 2*). Statistical heterogeneity was low (*I²* = 0%).

Meta-analysis of six observational studies—including 45 554 patients and 610 SSIs, (SSI rate 1.3%)—showed a statistically significant decrease in SSIs associated with weight-based dosing of SAP (RD −1.93 (−2.84 to −1.02)%; 95% PI −3.55 to −0.31%) (*Fig. 2*), with most studies focusing on orthopaedic surgery. Statistical heterogeneity was low (*I²* = 0%).

Overall, across the nine studies (45 657 patients and 611 SSIs, SSI rate 1.3%) weight-based dosing of cefazolin was associated with a significant reduction in SSIs compared with standard dosing (RD −1.86 (−2.61 to −1.12)%; 95% PI −3.22 to −0.51) (*Fig. 2*). Statistical heterogeneity was low (*I^2^* = 0%).

A sensitivity analysis using the arcsine difference model showed a difference of −0.06 (95% c.i. −0.08 to −0.04; 95% PI −0.08 to −0.04) favouring weight-based dosing of SAP (*Fig. S1*), which is consistent with the primary analysis. Statistical heterogeneity was low (*I^2^* = 5.4%).

A sensitivity analysis with the use of cefazolin only (without metronidazole) showed a statistically significant decrease in SSIs associated with weight-based dosing of SAP (RD −1.99 (−3.12 to −0.86)%; 95% PI −3.96 to −0.01%) (*Fig. S2*). Statistical heterogeneity was low (*I^2^* = 14%).

To evaluate both absolute and relative perspectives of the effect while preserving the clinical interpretability of the primary results, an additional analysis using the OR showed an overall statistically significant effect, with an OR of 0.45 (95% c.i. 0.34 to 0.60; 95% PI 0.28 to 0.73) favouring weight-based dosing of SAP (*Fig. S3*). Statistical heterogeneity was low (*I^2^* = 0.4%).

### Systematic review of all 33 included studies

**Fig. 3:**
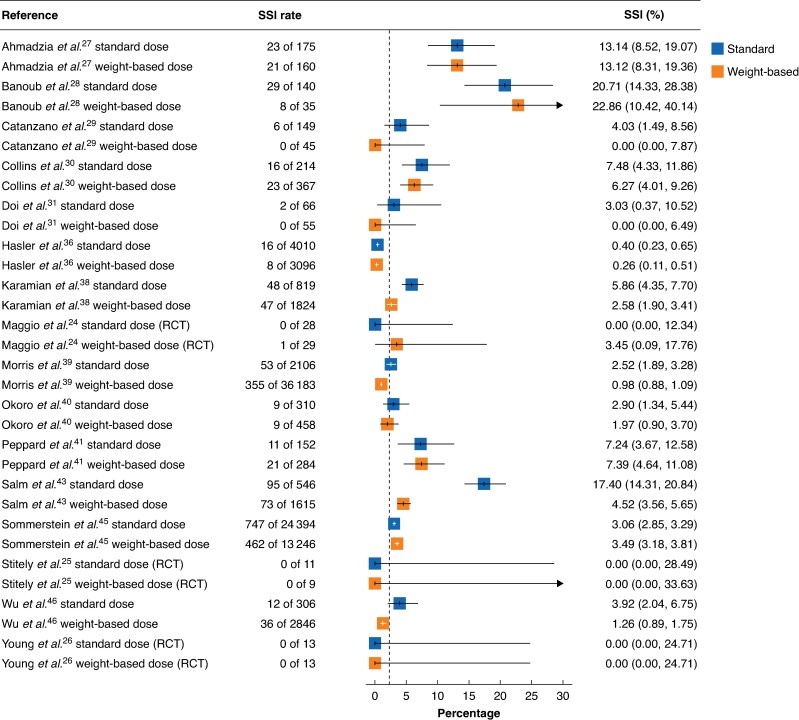
SSI rates after administration of weight-based *versus* standard surgical antibiotic prophylaxis (various antibiotic regimens) Surgical site infection (SSI) rates are shown with 95% confidence intervals.

An overview of reported SSI rates with 95% confidence intervals in observational comparative studies of weight-based dose *versus* standard dose is shown in *Fig. 3*. Ten^27,29–31,36,38–40,43,46^ of 16 studies showed a reduction in SSI rates using weight-based SAP compared with standard dosing. Summarized SSI data with 95% confidence intervals from studies that included SAP with cefazolin can be found in *Fig. S4*. Those from studies using other types of SAP in patients with overweight and obesity (weight ≥ 80 kg or BMI ≥ 30 kg/m^2^) are shown in *Fig. S5*: cefotetan and cefoxitin (*Fig. S5a*), cefuroxime (*Fig. S5b*), cefuroxime and metronidazole (*Fig. S5c*), vancomycin (*Fig. S5d*), and cefazolin and metronidazole (*Fig. S5e*). SSI rates varied from 0 to 22.86 (95% c.i. 0 to 40.14)%.

### Quality assessment

Overall scores determined using the revised cochrane risk-of-bias tool for randomized trials and the risk of bias in non-randomized studies—of interventions assessment tool for observational studies are shown in *Table 1*. The assessment for bias in the RCTs revealed ‘some concerns’ for one study^24^ and ‘low’ for two studies^25,26^. For the observational studies, the risk-of-bias analysis showed heterogeneity, and the assessment varied between ‘some concerns’ and ‘serious’. The scores on all seven subsets can be found in *Table S3*.

### GRADE assessment

Two GRADE assessments were carried out. The GRADE assessment of included RCTs revealed a very low certainty of evidence (*Table 2*). The starting certainty of evidence was high. As the risk of bias was considered ‘low’ in two studies and having ‘some concerns’ in one, no downgrading was needed. No downgrading was necessary for inconsistency (*I^2^* = 0%, τ^2^ = 0). The included RCTs involved only obstetric surgery with female patients, rather than a broader surgical population with patients of both sexes. One level of downgrading was needed for indirectness as pathogen, and tissue penetrations are different across male and female patients. Because there were very few events and the confidence intervals overlapped the thresholds of interest, two levels of downgrading were needed for imprecision. One level of downgrading was needed for publication bias, because the evidence was derived from a small number of small studies. In total, four levels of downgrading resulted in a very low certainty of evidence.

**Table 2:** GRADE assessment of RCTs and observational studies included in the meta-analysis

Outcome	Certainty assessment							SSI rate		Effect	Certainty
	No of studies	Study design	Risk of bias	Inconsistency	Indirectness	Imprecision	Publication bias	Weight-based dosing	Standard dosing	Risk Difference*	
SSI	3	RCT	Not serious	Not serious	Serious (−1 downgrade)†	Serious (−2 downgrade)‡	Serious (−1 downgrade)§	1 of 51 (2.0%)	0 of 52 (0%)	2.02 (−3.15, 7.19)	⨁◯◯◯ Very low
SSI	6	Observational	Serious (−1 downgrade)¶	Not serious	Not serious	Not serious	Serious (−1 downgrade)§	470 of 41 733 (1.1%)	140 of 3821 (3.7%)	−1.93 (−2.84, −1.02)	⨁◯◯◯ Very low

Starting certainty is high for randomized clinical trials (RCTs) and low for observational studies. Values are *n* (%) unless stated otherwise; *values in parentheses are 95% confidence intervals. †Included only obstetric patients; expected differences in pathogen and tissue penetration across male and female patients. ‡Few events and overlapping confidence intervals. §Small number of studies. ¶High risk of bias for all studies. GRADE, Grading of Recommendations, Assessment, Development and Evaluation; SSI, surgical site infection.

The GRADE assessment for observational studies resulted in a very low certainty of evidence (*Table 2*). The starting certainty of evidence was low, as all included studies in the meta-analysis were observational cohort studies. One level of downgrading was needed for risk of bias as all included studies were assessed as being at high risk of bias. No downgrading was needed for inconsistency because heterogeneity was low (*I^2^* = 0%). All but one study included only orthopaedic surgery rather than a broader surgical population. A sensitivity analysis excluding the study using metronidazole alongside cefazolin was undertaken, with results comparable to those of the primary analysis (RD −1.99 (95% c.i. −3.12 to −0.86)%; 95% PI −3.96 to −0.01%). As tissue penetrations are expected to be comparable, no downgrading was needed for indirectness. The confidence intervals did not overlap the thresholds of interest, so no downgrade was necessary for imprecision. Rating down one level for publication bias was needed because the evidence was from a small number of studies. In total, two levels of downgrading resulted in a very low certainty of evidence. An overview of the evaluation and considerations is available in *Table S4*.

## Discussion

This systematic review evaluated the effect of weight-based dosing of SAP on the incidence of SSI in patients with overweight and obesity. Meta-analysis of 3 RCTs comprising 103 patients with only 1 SSI showed no significant benefit in SSI reduction for weight-based SAP of cefazolin compared with standard dosing. However, meta-analysis of 6 observational studies comprising 45 554 patients suggested that weight-based dosing of cefazolin may be associated with lower SSI rates in patients with overweight and obesity, with most studies focusing on orthopaedic surgery. A sensitivity analysis excluding one study^30^ that used metronidazole in addition to cefazolin in elective colorectal surgery showed similar results. Descriptive statistics for all studies using different antibiotic regimens showed low SSI rates, and it was found that weight-based dosing of SAP resulted in lower SSI rates in 10 of 16 studies.

A recently conducted systematic review by Coates *et al.*^17^ investigated whether a 2-g dose of cefazolin is sufficient to achieve adequate plasma and tissue concentrations in patients with obesity undergoing surgery for up to 4 hours. They reported that 9 of 15 studies provided evidence for meeting the MIC with a 2-g dose of cefazolin, and concluded that there is no need to increase the dose beyond 2-g in patients with obesity^17^. However, MIC targets varied between the included studies, as did the BMI ranges of the groups under comparison as well as the tissue sampled, rendering the data difficult to interpret. Importantly, these findings contrast with those in an earlier systematic review by Fischer *et al.*^18^ that found evidence to support the use of an increased dose of cefazolin in patients weighing ≥ 120 kg to prevent SSI, and existing guidelines state that the dose of cefazolin should be increased to 2-g for patients weighing ≥ 80 kg and to 3-g for those weighing ≥ 120 kg, considering the favourable safety profile of cefazolin. Since the issue of these guidelines and Fischer’s systematic review, a substantive amount of new research has been conducted to explore different dosing strategies to prevent SSI in patients with overweight and obesity. The findings of Coates *et al*.^17^ casted doubt on the status quo. The present findings now indicate that new evidence is in line with the existing guidelines, and further strengthens the recommendation for weight-based dosing.

This systematic review is limited by the lack of high-quality evidence for the primary outcome, SSI. The included RCTs were severely underpowered, enrolling only 103 patients with a single SSI event, as they were designed to assess sample or tissue concentrations rather than clinical outcomes. Consequently, the data are insufficient to determine an effect on SSI. Although observational studies contributed evidence, their clinical and methodological heterogeneity restricted the strengths of the conclusions. Substantial variation was noted between studies in types of antibiotic used and their administration. For example, some studies used first-generation cephalosporins and others second-generation cephalosporins. Moreover, variability in timing of administration and redosing of antibiotic(s) during surgery, different dosing within a single weight category, and comparing a set dose between weight categories in different study designs (for example single-arm design, comparative design), prevented standardization of data. The criteria used to define overweight and obesity also varied markedly among studies, with different cut-off values used. Some studies used inclusion criteria of a bodyweight of ≥ 80 kg, which did not specifically encompass patients with obesity. BMI and total bodyweight were used interchangeably to define obesity, and the lack of a standardized definition of obesity is a major limitation of the available studies. Moreover, there was inconsistency in reporting use of the CDC classification, and information on CDC categories was missing from some reports. Many studies did not include SSI rates as the primary outcome. In some instances, authors simply reported that there were no SSIs or provided numerical data without subsequent analysis. Additionally, some studies did not report SSI diagnostics and follow-up, which may have resulted in loss of relevant SSI data.

Another limitation is the frequently missing data. Several studies^58–65^ were excluded from this review owing to lack of reporting on SSI as outcome parameter. Among the included studies, one^44^ did not specify which study arm the SSI occurred in and was therefore excluded from the meta-analysis.

In addition to clinical and methodological heterogeneity, another limitation is the insufficient evidence on potential adverse effects or toxicity of higher SAP doses in patients with obesity. A recent systematic review^66^ stated that there is absence of robust pharmacokinetic data to inform dose adjustments, and the potential toxicity of altered dosing strategies should be considered.

Overall, clinical and methodological heterogeneity (for example definitions and interventions) in the available studies limited the reliability of the summary data. This systematic review primarily reflects the limitations of the existing literature, and no definite clinical recommendations can be made from the existing data. Despite these limitations, considering consensus guidelines, the observed effect (albeit with uncertainty), and the biological rationale, it is unlikely that higher-quality data will emerge from sufficiently powered RCTs^9^. As such, the focus of future studies may shift towards optimizing dosing strategies through a better understanding of pharmacokinetics and safety profiles of higher doses of SAP in patients with obesity, with SSI data structured according to MIC targets achieved.

Based on data from observational studies, the findings of this review suggest that the use of weight-based dosing of SAP may reduce the risk of SSI in patients with obesity compared with standard dosing of SAP, but the existing evidence is very uncertain.

## Supplementary Material

zrag015_Supplementary_Data

## Data Availability

Data can be provided upon request and in agreement of terms. No individual-participant data were used.
