# Supplementary material for: Weight-based dosing of surgical antibiotic prophylaxis in patients with obesity: meta-analysis
Source: BJS Open. 2026 Apr 17;10(2):zrag015. doi: 10.1093/bjsopen/zrag015 (PMC13089643; doi:10.1093/bjsopen/zrag015)

**Title:** Weight-based dosing of surgical antibiotic prophylaxis in patients with obesity**:** A Systematic Review and Meta-Analysis

**Authors:** Hiske Huisman MD,^1,2^ Karlijn Huinink MSc,^1,2^ Nathan Bontekoning MD,^1,2^ Stijn W. de Jonge MD, PhD,^1,2^ Gerjon Hannink, PhD,^3*^ prof. Paulina Salminen, MD, PhD,^4,5*^ prof. Marja A. Boermeester MD, PhD^1,2*^ ^*^ Shared last authorship; these authors contributed equally to this article

**Affiliations:**

^1^ Amsterdam UMC location University of Amsterdam, Department of Surgery, Meibergdreef 9, Amsterdam, The Netherlands
^2^ Amsterdam Gastroenterology Endocrinology & Metabolism, Amsterdam, The Netherlands

^3^ Radboud University Medical Center, Department of Medical Imaging, Nijmegen, The Netherlands

^4^ Division of Digestive Surgery and Urology, Turku University Hospital, Turku, Finland

^5^ Department of Surgery, University of Turku, Turku, Finland

**Corresponding author:** Prof. M.A. Boermeester, Amsterdam UMC location University of Amsterdam, Department of Surgery, Meibergdreef 9, Amsterdam, the Netherlands, e-mail: m.a.boermeester@amsterdamumc.nl, telephone number +31205669111

**Supplementary Materials – Index**

**Supplementary Methods**

Search strategy *page 2-4*

**Supplementary Figures and Tables**

Table S1: Reasons for full text exclusion *page 5-6*

Table S2: Complete table of study characteristics *page 7-10*

Table S3: Cochrane risk of bias *page 11-12*

Table S4: GRADE assessment *page 13-15*

Figure S1: additional figures *page 16*

Figure S2: additional figures *page 16*

Figure S3: additional figures *page 17*

Figure S4: additional figures *page 18*

Figure S5: additional figures *page 19*

**Supplementary methods.** Search strategy.

Search strategies: Faridi Jamaludin, Amsterdam UMC location University of Amsterdam, Medical Library AMC, Meibergdreef 9, Amsterdam, The Netherlands

**3-9-2024**:

| Databases: | Before deduplication | After deduplication* |
| --- | --- | --- |
| PubMed  Embase (Ovid)  Cochrane CDSR en CENTRAL (Wiley)  CINAHL (Ebsco) |  |  |
| Total | 2481 | 1760 |

*Lobbestael, G. (2023). DedupEndNote (Version 1.0.0) [Computer software]. https://github.com/globbestael/DedupEndNote

Pubmed

602 results

("Surgical Wound Infection"[Mesh] OR ("Postoperative Complications"[Mesh] AND "Wound Infection"[Mesh]) OR surgical site infection*[tiab] OR SSI[tiab] OR SSIs[tiab] OR surgical wound infection*[tiab] OR surgical infection*[tiab] OR post-operative wound infection*[tiab] OR postoperative wound infection*[tiab] OR post-operative infection*[tiab] OR postoperative infection*[tiab])

AND

("Antibiotic Prophylaxis"[Mesh] OR "Anti-Bacterial Agents"[Mesh] OR "Cefuroxime"[Mesh] OR "Metronidazole"[Mesh] OR "Cefazolin"[Mesh] OR "Levofloxacin"[Mesh] OR "Clindamycin"[Mesh] OR "Vancomycin"[Mesh] OR "Ciprofloxacin"[Mesh] OR "Ampicillin"[Mesh] OR "Aztreonam"[Mesh] OR "Cefotaxime"[Mesh] OR "Cefoxitin"[Mesh] OR "Cefotetan"[Mesh] OR "Ceftriaxone"[Mesh] OR "ertapenem" [Supplementary Concept] OR "Fluconazole"[Mesh] OR "Gentamicins"[Mesh] OR "moxifloxacin" [Supplementary Concept] OR "piperacillin, tazobactam drug combination" [Supplementary Concept] OR "sultamicillin" [Supplementary Concept] OR "Sulbactam"[Mesh] OR "Erythromycin"[Mesh] OR "Neomycin"[Mesh] OR antibacterial agent*[tiab] OR antimicrobial[tiab] OR antibiotic*[tiab] OR antiinfective agent* [tiab] OR cefuroxime[tiab] OR metronidazole[tiab] OR cefazolin[tiab] OR levofloxacin[tiab] OR clindamycin[tiab] OR vancomycin[tiab] OR ciprofloxacin[tiab] OR ampicillin[tiab] OR aztreonam[tiab] OR cefotaxime[tiab] OR cefoxitin[tiab] OR cefotetan[tiab] OR ceftriaxone[tiab] OR ertapenem[tiab] OR fluconazole[tiab] OR gentamicin[tiab] OR moxifloxacin[tiab] OR piperacillin-tazobactam[tiab] OR ampicillin-sulbactam[tiab] OR erythromycin[tiab] OR neomycin[tiab])

AND

("Body Weight"[Mesh] OR "Obesity"[Mesh] OR "Overweight"[Mesh] OR overweigh*[tiab] OR obes*[tiab] OR morbid-obes*[tiab] OR weight-based[tiab] OR weight-adjusted[tiab] OR weight-adopted[tiab] OR patients weigh*[tiab] OR body weigh*[tiab] OR adipos*[tiab] OR corpulen*[tiab])

EMBASE (Ovid)

Database(s): Embase Classic+Embase 1947 to 2024 August 30
Search Strategy:

| # | Searches | Results |
| --- | --- | --- |
| 1 | surgical infection/ or "postoperative infection"/ | 110638 |
| 2 | ((surgical or postoperative or post-operative or peri-operative or perioperative) adj3 infection*).ti,ab,kf. | 60102 |
| 3 | (SSI or SSIs).ti,ab,kf. | 17310 |
| 4 | 1 or 2 or 3 | 138413 |
| 5 | *antibiotic prophylaxis/ or exp *antiinfective agent/ or *cefuroxime/cb, cr, dt, pk or *metronidazole/cb, cr, dt, pk or *cefazolin/cb, cr, dt, pk or *levofloxacin/cb, cr, dt, pk or *clindamycin/cb, cr, dt, pk or *vancomycin/cb, cr, dt, pk or *ciprofloxacin/cb, cr, dt, pk or *ampicillin/cb, cr, dt, pk or *aztreonam/cb, cr, dt, pk or *cefotaxime/cb, cr, dt, pk or *cefoxitin/cb, cr, dt, pk or *cefotetan/cb, cr, dt, pk or *ceftriaxone/cb, cr, dt, pk or *ertapenem/cb, cr, dt, pk or *fluconazole/cb, cr, dt, pk or *gentamicin/cb, cr, dt, pk or *moxifloxacin/cb, cr, dt, pk or *piperacillin plus tazobactam/cb, cr, dt, pk or *sultamicillin/cb, cr, dt, pk or *sulbactam/cb, cr, dt, pk or *erythromycin/cb, cr, dt, pk or *neomycin/cb, cr, dt, pk | 2052032 |
| 6 | (antibacterial agent* or antimicrobial or antibiotic* or antiinfective agent* or cefuroxime or metronidazole or cefazolin or levofloxacin or clindamycin or vancomycin or ciprofloxacin or ampicillin or aztreonam or cefotaxime or cefoxitin or cefotetan or ceftriaxone or ertapenem or fluconazole or gentamicin or moxifloxacin or piperacillin-tazobactam or ampicillin-sulbactam or erythromycin or neomycin).ti,ab,kf. | 998281 |
| 7 | 5 or 6 | 2629594 |
| 8 | exp obesity/ or exp body weight/ | 1352950 |
| 9 | (overweigh* or obes* or morbid-obes* or weight-based or weight-adjusted or weight-adopted or patients weigh* or body weigh* or adipos* or corpulen*).ti,ab,kf. | 1093266 |
| 10 | 8 or 9 | 1709299 |
| 11 | 4 and 7 and 10 | 1296 |

Cochrane Library

[Cochrane Central Register of Controlled Trials](https://www.cochranelibrary.com/)

Issue 8 of 12, August 2024

[Cochrane Database of Systematic Reviews](https://www.cochranelibrary.com/)

Issue 9 of 12, September 2024

ID Search Hits

#1 ((surgical or postoperative or post-operative or peri-operative or perioperative) near/3 infection*):ti,ab,kw 11903

#2 (SSI or SSIs):ti,ab,kw 2275

#3 #1 or #2 12546

#4 (antibacterial agent* or antimicrobial or antibiotic* or antiinfective agent* or cefuroxime or metronidazole or cefazolin or levofloxacin or clindamycin or vancomycin or ciprofloxacin or ampicillin or aztreonam or cefotaxime or cefoxitin or cefotetan or ceftriaxone or ertapenem or fluconazole or gentamicin or moxifloxacin or piperacillin-tazobactam or ampicillin-sulbactam or erythromycin or neomycin):ti,ab,kw 67044

#5 (overweigh* or obes* or morbid-obes* or weight-based or weight-adjusted or weight-adopted or patients weigh* or body weigh* or adipos* OR corpulen*):ti,ab,kw 165761

#6 #3 and #4 and #5 in Cochrane Reviews, Trials 338

CINAHL (Ebsco)

245 results

(MH "Surgical Wound Infection") OR ( (MH "Postoperative Complications") AND (MH "Wound Infection") ) OR ( TI ( (surgical or postoperative or post-operative or peri-operative or perioperative) N3 infection* ) OR AB ( (surgical or postoperative or post-operative or peri-operative or perioperative) N3 infection* ) OR TI ( SSI or SSIs ) OR AB ( SSI or SSIs ) )

AND

(MH "Antibiotic Prophylaxis") OR (MH "Antiinfective Agents+") OR ( TI ( antibacterial agent* or antimicrobial or antibiotic* or antiinfective agent* or cefuroxime or metronidazole or cefazolin or levofloxacin or clindamycin or vancomycin or ciprofloxacin or ampicillin or aztreonam or cefotaxime or cefoxitin or cefotetan or ceftriaxone or ertapenem or fluconazole or gentamicin or moxifloxacin or piperacillin-tazobactam or ampicillin-sulbactam or erythromycin or neomycin ) OR AB ( antibacterial agent* or antimicrobial or antibiotic* or antiinfective agent* or cefuroxime or metronidazole or cefazolin or levofloxacin or clindamycin or vancomycin or ciprofloxacin or ampicillin or aztreonam or cefotaxime or cefoxitin or cefotetan or ceftriaxone or ertapenem or fluconazole or gentamicin or moxifloxacin or piperacillin-tazobactam or ampicillin-sulbactam or erythromycin or neomycin ) )

AND

( (MH "Body Weight+") OR (MH "Obesity+") ) OR TI ( overweigh* or obes* or morbid-obes* or weight-based or weight-adjusted or weight-adopted or patients weigh* or body weigh* or adipos* or corpulen*) OR AB ( overweigh* or obes* or morbid-obes* or weight-based or weight-adjusted or weight-adopted or patients weigh* or body weigh* or adipos* or corpulen*)

**UPDATE SEARCH 3-9-2024 – 21-10-2025**

**21-10-2025:**

| Databases: | Before deduplication | After deduplication* |
| --- | --- | --- |
| PubMed  Embase (Ovid)  Cochrane CDSR en CENTRAL (Wiley)  CINAHL (Ebsco) |  |  |
| Total | 301 | 243 |

*Lobbestael, G. (2025). DedupEndNote (Version 1.1.0) [Computer software]. https://github.com/globbestael/DedupEndNote

**Supplementary Figures and Tables**

**Table S1.** Reasons for exclusion

|  | **Study** | **Reason for exclusion** |
| --- | --- | --- |
| 1. | Abdel Halim et al. (2024)^1^ | No SSI as outcome parameter |
| 2. | Abdel Jalil et al. (2017)^2^ | No SSI as outcome parameter |
| 3. | Badge et al. (2022)^3^ | No subgroup data available |
| 4. | Bhargava et al. (2024)^4^ | No subgroup data available |
| 5. | Bindellini et al. (2024)^5^ | No SSI as outcome parameter |
| 6. | Chopra et al. (2012)^6^ | SSI cases were matched to controls a-priori (ratio 1:3) |
| 7. | Cies et al. (2012)^7^ | Weight-based dosing was applied in children, but no obese patients were included |
| 8. | Freeman et al. (2011)^8^ | No subgroup data available |
| 9. | Gordon et al. (2006)^9^ | Opinion article |
| 10. | Grégoire et al. (2018)^10^ | No SSI as outcome parameter |
| 11. | Ho et al. (2012)^11^ | No SSI as outcome parameter |
| 12. | Housman et al. (2022)^12^ | No SSI as outcome parameter |
| 13. | La Rosa et al. (2019)^13^ | No subgroup data available |
| 14. | Olinger et al. (2018)^14^ | No subgroup data available |
| 15. | Palma et al. (2018)^15^ | No SSI as outcome parameter |
| 16. | Swank et al. (2015)^16^ | No SSI as outcome parameter |

**References**

1. Abdel Halim AS, Ali MAM, Al Mamari R, Al Raisi F, Boufahja F, Chaudhary AA, et al. A Retrospective Exploration of Pre-operative Antibiotic Prophylaxis with Cefazolin in Cesarean Sections: Implications for Obstetrics and Gynecologic Surgery. Surg Infect (Larchmt). 2024;25(7):513–20. Doi.10.1089/sur.2024.048

2. Abdel Jalil MH, Abu Hammour K, Alsous M, Awad W, Hadadden R, Bakri F, et al. Surgical site infections following caesarean operations at a Jordanian teaching hospital: Frequency and implicated factors. Sci Rep. 2017;7(1):12210. Doi.10.1038/s41598-017-12431-2

3. Badge H, Churches T, Xuan W, Naylor JM, Harris IA. Timing and duration of antibiotic prophylaxis is associated with the risk of infection after hip and knee arthroplasty. Bone & Joint Open. 2022;3(3):252–60. Doi.10.1302/2633-1462.33.Bjo-2021-0181.R1

4. Suveer Bhargava, Ravindra Singh, Banga P. To compare the effectiveness of a single dose of preventive antibiotic with the use of empirical post-operative antibiotics in preventing Surgical Site Infection (SSI). International Journal of Life Sciences, Biotechnology and Pharma Research. 2024; Vol. 13(No. 2):288–93. Doi.10.69605

5. Bindellini D, Simon P, Busse D, Michelet R, Petroff D, Aulin LBS, et al. Evaluation of the need for dosing adaptations in obese patients for surgical antibiotic prophylaxis: a model-based analysis of cefazolin pharmacokinetics. Br J Anaesth. 2025;134(4):1041–9. Doi.10.1016/j.bja.2024.11.044

6. Chopra T, Marchaim D, Lynch Y, Kosmidis C, Zhao JJ, Dhar S, et al. Epidemiology and outcomes associated with surgical site infection following bariatric surgery. Am J Infect Control. 2012;40(9):815–9. Doi.10.1016/j.ajic.2011.10.015

7. Cies JJ, Chan S, Hossain J, Brenn BR, Di Pentima MC. Influence of body mass index and antibiotic dose on the risk of surgical site infections in pediatric clean orthopedic surgery. Surg Infect (Larchmt). 2012;13(6):371–6. Doi.https://doi.org/10.1089/sur.2011.096

8. Freeman JT, Anderson DJ, Hartwig MG, Sexton DJ. Surgical site infections following bariatric surgery in community hospitals: a weighty concern? Obes Surg. 2011;21(7):836–40. Doi.10.1007/s11695-010-0105-3

9. Gordon SM. Antibiotic prophylaxis against postoperative wound infections. Cleve Clin J Med. 2006;73 Suppl 1:S42–S5. Doi.http://dx.doi.org/10.3949/ccjm.73.suppl_1.s42

10. Gregoire M, Dumont R, Ronchi L, Woillard JB, Atthar V, Letessier E, et al. Prophylactic cefazolin concentrations in morbidly obese patients undergoing sleeve gastrectomy: do we achieve targets? Int J Antimicrob Agents. 2018;52(1):28–34. Doi.10.1016/j.ijantimicag.2018.02.015

11. Ho VP, Nicolau DP, Dakin GF, Pomp A, Rich BS, Towe CW, et al. Cefazolin dosing for surgical prophylaxis in morbidly obese patients. Surg Infect (Larchmt). 2012;13(1):33–7. Doi.10.1089/sur.2010.097

12. Housman ST, McWhorter PB, Barie PS, Nicolau DP. Ertapenem Concentrations in Obese Patients Undergoing Surgery. Surg Infect (Larchmt). 2022;23(6):545–9. Doi.10.1089/sur.2022.005

13. La Rosa M, Omere C, Redfern T, Abdelwahab M, Spencer N, Villarreal J, et al. The impact of low-dose versus high-dose antibiotic prophylaxis regimens on surgical site infection rates after cesarean delivery. Archives of Gynecology and Obstetrics. 2019;301(1):69–73. Doi.10.1007/s00404-019-05370-y

14. Olinger CR, Carver DC, Nolan VG, Weinlein JC. The Effect of Under-Dosing Prophylactic Antibiotics in the Care of Open Tibial Fractures. J Orthop Trauma. 2018;32(7):322–6. Doi.10.1097/BOT.0000000000001171

15. Palma EC, Meinhardt NG, Stein AT, Heineck I, Fischer MI, de Araujo B, et al. Efficacious Cefazolin Prophylactic Dose for Morbidly Obese Women Undergoing Bariatric Surgery Based on Evidence from Subcutaneous Microdialysis and Populational Pharmacokinetic Modeling. Pharm Res. 2018;35(6):116. Doi.10.1007/s11095-018-2394-5

16. Swank ML, Wing DA, Nicolau DP, McNulty JA. Increased 3-gram cefazolin dosing for cesarean delivery prophylaxis in obese women. Am J Obstet Gynecol. 2015;213(3):415 e1–8. Doi.10.1016/j.ajog.2015.05.030

**Table S2.** Complete table 1

| Study design | Author (year) | N | Specialty | CDC-level | Weight categories (n) | Type of SAP | Dosing (n) | Primary outcome | SSI/n (%) | Statistics | Follow-up | Risk of Bias |
| --- | --- | --- | --- | --- | --- | --- | --- | --- | --- | --- | --- | --- |
| RCT | Maggio et al.  (2015) | 57 | Obstetrics | II | BMI ≥ 30 | Cefazolin | I: 3 g (29) II: 2 g (28) | Adipose tissue concentration | I: 1/29 (3.4) II: none | χ2: n.s. | 6-8 weeks | Some concerns |
|  | Stitely et al.  (2023) | 20 | Obstetrics | II | BMI ≥ 35 | Cefazolin | I: 4 g (9) II: 2 g (11) | Plasma concentration | None | NA | 6-8 weeks | Low |
|  | Young et al.  (2015) | 26 | Obstetrics | II | BMI ≥ 30 | Cefazolin | I: 3 g (13) II: 2 g (13) | Plasma concentration | None | NA | NI | Low |
| Observational *Comparative* | Ahmadzia et al. (2015) | 335 | Obstetrics | II | ≥ 290 pounds | Cefazolin | I: 3 g (160) II: 2 g (175) | SSI | I: 21/160 (13.1) II: 23/175 (13.1) | Adjusted OR 1.33 (CI 95% 0.64–2.74) | 30 days | Serious |
|  | Banoub et al.  (2018) | 175 | General, obstetrics | I-II | ≥ 120 kg | Cefotetan/cefoxitin | I: 3 g (35) II: 2 g (140) | SSI | I: 8/35 (22.9) II: 29/140 (20.7) | Univariate analysis RR 1.10 (CI 95% 0.55–2.20);  p = 0.78 | 30 days | Some concerns |
|  | Catanzano et al. (2014) | 216 | Orthopaedics  (Spine surgery) | I | NI | Vancomycin | Underdosed:  < 1 g (149)  Appropriate:  1 g (45) Overdosed:  > 1 g (22) | Pharmacokinetics | Underdosed: 6/149 (4.0) Appropriate: none Overdosed: none | NA | NI | NI |
|  | Collins et al. (2025) | 581 | Colorectal surgery | II-III | ≥ 120 kg | Cefazolin / metronidazole | I: 3g + 500 mg (367)  II: 2g + 500mg (214) | SSI | I: 23/367 (6.3)  II: 16/214 (7.5) | χ2: I vs. II (p = 0.574) | 30 days | Serious |
|  | Doi et al. (2024) | 121 | Orthopaedics |  | ≥ 80 kg | Cefazolin | I: 2 g (55)  II: 1 g (66) | SSI | I: 0/55  II: 2/66 (3.0) | Fisher’s exact test  (p = 0.295) | 30 days | Serious |
|  | Ferraz et al.  (2003) | 363 | Bariatric surgery | II | BMI ≥ 40 | I: Ampicillin/  sulbactam II: Ceftriaxone | I: 3 g (83) II: 1 g (280) | SSI | I: 5/83 (6.0)  II:19/280 (6.8) | χ2: 0.06;  p = 0.8 | 30 days | Serious |
|  | Ferraz et al.  (2015) | 896 | Bariatric surgery | II | BMI ≥ 40 | I: Ampicillin/  sulbactam II: Ertapenem III: Cefazolin | I: 2 g/1 g (194) II: 1 g (303) III: 2 g (399) | SSI | I: 8/194 (4.1) II: 6/303 (2.0) III: 6/399 (1.5) | χ2: I vs. II  (p = 0.37);  I vs III (p = 0.14); II vs III (p = 0.89) | Min. 30 days | Serious |
|  | Ferrer Pomares et al. (2024) | 84 | Orthopaedics  (Spine surgery) | I | BMI ≥ 30 | A: Cefazolin B: Cefazolin +  Amikacin  C: Cefazolin + amikacin (every 8h for 74h) | A: 2 g (30) B: 2 g + 500 mg (30)  C: 2 g + 500 mg (24) | SSI | A: 8/30 (26.7) B: 4/30 (13.3)  C: 2/24 (8.3) | NA | 90 days | Some concerns |
|  | Fouks et al.  (2020) | 42 | Obstetrics | II | I: ≥ 80 kg (21) II: < 80 kg (21) | Cefazolin | I: 2 g (21) II: 1 g (21) | Plasma levels | None | NA | NI | NI |
|  | Hasler et al.  (2021) | 7,106 | Orthopaedics  (General procedures) | I | ≥ 80 kg | Cefuroxime | I: 3 g (3,096) II: 1.5 g (4,010) | SSI | I: 8/3,096 (0.3) II: 16/4,010 (0.4) | NA | 30 days/  1 year (implant-related) | Serious |
|  | Hopkins et al.  (2023) | 1,273 | Obstetrics | II | BMI ≥ 40 | Cefazolin  Cefazolin + azithromycin | I: 3 g (303) II: 3 g + 500 mg (970) | SSI | I: 31/303 (10.2) II: 65/970 (6.7) | χ2; p = 0.04 | NI | Some concerns |
|  | Karamian et al. (2022) | 2,643 | Orthopaedics  (Spine surgery) | I | A: < 60 kg (258)  B: 60-120 kg (2,194)  C: ≥ 120 kg (191) | Cefazolin | Recommended dose: 1 g if < 60 kg, 2 g if 60-120 kg and 3 g if ≥ 120 kg (1,824) Underdosed: 819 | SSI | Recommended dose: 47/1,824 (2.6) Underdosed: 48/819 (5.9) | Odds Ratio (OR): 0.45;  p <0.001 | NI | Serious |
|  | Morris et al.  (2020) | 38,289 | Orthopaedics  (Hip and knee arthroplasties) | I | A: < 80 kg (15,114)  B: 80-120 kg (21,164)  C: ≥ 120 kg (2,011) | Cefazolin | Recommended dose: 1 g if < 80 kg, 2 g if 80-120 kg and 3 g if ≥ 120 kg (36,183) Underdosed: (2,106) | SSI | Recommended dose: 355/36,183 (1.0) Underdosed: 53/2,106 (2.5) | Multivariable analysis (excluding weight) OR for SSI 2.19 (CI 95% 1.61-2.99);  p < 0.001 | 90 days | Serious |
|  | Okoro et al.  (2023) | 768 | Orthopaedics  (Hip and knee arthroplasties) | I | NI | Cefazolin | New Regime: 2 g if < 120 kg and 3 g if ≥ 120 kg (458) Old Regime: 2 g (310) | SSI | New Regime: 9/458 (2.0) Old Regime: 9/310 (2.9) | Weighted dataset RR 0.66 (CI 95% 0.32-1.40);  p = 0.27 | 2 years | Serious |
|  | Peppard et al. (2017) | 436 | Neuro,  0rthopaedics, general or emergency trauma | I-III | ≥ 100 kg | Cefazolin | I: 3 g (284) II: 2 g (152) | SSI | I: 21/284 (7.4) II: 11/152 (7.2) | Adjusted OR 0.87 (CI 95% 0.36–2.06);  p = 0.77 | Max. 90 days | Some concerns |
|  | Perez et al. (2024) | 816 | Obstetrics | II | BMI ≥ 30 | I: cefazolin  II: cefazolin + azithromycin |  | SSI | I: 25/525 (4.8)  II: 6/291 (2.1) | Unadjusted OR: 0.42 (CI 95% 0.14-1.07) | NI | Some concerns |
|  | Salm et al.  (2020) | 2,161 | Visceral, vascular, orthopaedics or trauma* | I-III | ≥ 80 kg | Cefuroxime + metronidazole | Double-dose:  3 g + 1g (1,615) Single-dose:  1.5 g + 500 mg (546) | SSI | Double-dose: 73/1,615 (4.5) Single-dose: 95/546 (17.4) | OR: 4.4 (CI 95% 3.18-6.23);  p < 0.001 | 30 days | Some concerns |
|  | Scheck et al.  (2017) | 986 | Obstetrics | II | BMI ≥ 30 | Cefazolin | I: 3 g (731) II: 2 g (255) | SSI | NI | OR: 0.31 (CI 95% 0.14–0.69);  p = 0.03 | 30 days | Serious |
|  | Sommerstein et al. (2021) | 37,640 | Variousᵃ | I-III | ≥ 80 kg | Cefuroxime | I: 3 g (13,246) II: 1.5 g (24,394) | SSI | I: 462/13,246 (3.5) II: 747/24,394 (3.1) | Adjusted OR: 0.89 (CI 95%  0.78-1.02);  p = 0.10 | 30 days/  1 year (implant-related) | Some concerns |
|  | Wu et al.  (2016) | 3,152 | Orthopaedics  (Total knee arthroplasty) | I | NI | Cefazolin | Optimal dose: 2 g ≥ 80 kg and 1 g < 80 kg (2,846) Non-optimal dose: (306) | SSI | Optimal dose: 36/2,846 (1.3) Non-optimal dose:  12/306 (4.0) | Univariate analysis OR: 0.29 (CI 95% 0.17-0.62);  p < 0.01 | 30/1 year (deep infection) | Serious |
| *Single arm* | Belveyre et al. (2019) | 183 | Bariatric surgery | II | BMI ≥ 35 | Cefoxitin | 4 g (183) | Pharmacokinetics/pharmacodynamic target | 2/183 (1.1) ᵇ | NA | 30 days | Some concerns |
|  | Chen et al.  (2017) | 37 | Bariatric surgery | II | BMI ≥ 35 | Cefazolin | 2 g (37) | Serum levels | None | NA | 30 days | NI |
|  | Cinotti et al.  (2017) | 116 | Bariatric surgery | II | A: BMI 40 - 50 (79) B: BMI 50.1 - 65 (37) | Cefazolin | 4 g (116) | Tissue concentrations | None | NA | 30 days | Serious |
|  | Edmiston et al. (2004) | 38 | Bariatric surgery | II | A: BMI 40-49 (17)  B: BMI 50-59 (11)  C: BMI ≥ 60 (10) | Cefazolin | 2 g (38) | Serum concentrations | A: 3/17 (17.6) B: 1/11 (9.1) C: 3/10 (30) | χ2: n.s. | NI | Serious |
|  | Hites et al.  (2016) | 63 | Bariatric surgery | II | A: BMI <35 kg/m2 (20) B: BM I≥35 kg/m2 (43) | Cefazolin | 2g (63) | Serum concentrations | A: 0/20 B: 1/43 (2.3) | NA | 30 days | Serious |
|  | Hollis et al.  (2015) | 10 | Cardiology | I | ≥ 120 kg (1) | Cefazolin | 2 g (10) | Serum concentrations | None | NA | 30 days | NI |
|  | Hussain et al.  (2018) | 304 | General, gynaecology & obstetrics, orthopaedics (arthroplasty, ankle fracture) | I-IV | Non-obese: < 120 kg (152) Obese: ≥ 120 kg (152) | Cefazolin | 2 g (304) | SSI | Non-obese: 7/152 (4.6) Obese: 13/152 (8.6) | χ2: p = 0.25 | 90 days | Serious |
|  | Moine et al.  (2016) | 30 | Bariatric surgery | II | A: BMI ≥ 40 (25) B: BMI < 40 (5) | Cefoxitin | 40 mg/kg TBW | Serum concentrations | None | NA | NI | NI |
|  | Rodríguez  de Castro et al. (2020) | 49 | Trauma  (orthopaedic and non-implant trauma surgery) | II | Non-obese: < 100 kg or BMI < 30 (26) Obese: ≥ 100 kg or BMI ≥ 30 (23) | Cefazolin | 2 g (49) | SSI | Non-obese: 2/26 (7.7) Obese: 2/23 (8.7) | χ2: n.s. | 90 days | Serious |
|  | Unger et al.  (2014) | 195 | Various ᵃ | I-II | Non-obese: BMI < 30 (96) Obese: BMI ≥ 30 (99) | Cefazolin | 2 g (195) | SSI | Non-obese: 7/96 (7.3) Obese: 5/99 (5.1) | χ2: p = 0.56 | 30 days | Serious |

ᵃ Potentially including: bariatric, cardiology, general, gynaecology, neurosurgery, orthopaedics, plastics, podiatry, trauma or vascular

ᵇ One SSI occurred in 9 patients receiving 2 g cefoxitin, but these patients were excluded for analysis.

AB antibiotics; BMI body mass index; CDC centre for disease control and prevention; CI confidence interval; NA not applicable; NI not informed; n.s. not significant; OR odds ratio; RCT randomized controlled trial; RR risk ratio; SAP Surgical Antibiotic Prophylaxis; SSI surgical site infection; TBW total body weight

* Not clear if emergency trauma surgery was included

**Table S3.** Cochrane Risk of Bias

Bias in classification

Bias in selection results

Bias in measurement

Bias due to missing data

Deviation from intended intervention

Bias in selectionin study

Risk due to confounding

| **Study**  Ahmadzia (2015) |  | |  | |  | |  | |  | |  | |  | |  | | **Overall** | | |  | |
| --- | --- | --- | --- | --- | --- | --- | --- | --- | --- | --- | --- | --- | --- | --- | --- | --- | --- | --- | --- | --- | --- |
| Banoub (2018) |  | |  | |  | |  | |  | |  | |  | |  | |  | |  |  |  |
| Belveyre (2019) |  | |  | |  | |  | |  | |  | |  | |  | |  | |  |  |  |
| Catanzano (2014) |  | |  | |  | |  | |  | |  | |  | |  | |  | |  |  |  |
| Chen (2017) |  | |  | |  | |  | |  | |  | |  | |  | |  | | |  | |
| Cinotti (2017) |  | |  | |  | |  | |  | |  | |  | |  | |  | | |  | |
| Collins (2025) |  | |  | |  | |  | |  | |  | |  | |  | |  | | |  | |
| Doi (2025) |  | |  | |  | |  | |  | |  | |  | |  | |  | | |  | |
| Edmistion (2004) |  | |  | |  | |  | |  | |  | |  | |  | |  | | |  | |
| Ferraz (2003) |  | |  | |  | |  | |  | |  | |  | |  | |  | | |  | |
| Ferraz (2015) |  | |  | |  | |  | |  | |  | |  | |  | |  | | |  | |
| Ferrer Pomares (2024) |  | |  | |  | |  | |  | |  | |  | |  | |  | | |  | |
| Fouks (2020) |  | |  | |  | |  | |  | |  | |  | |  | |  | | |  | |
| Hasler (2020) |  | |  | |  | |  | |  | |  | |  | |  | |  | | |  | |
| Hites (2016) |  | |  | |  | |  | |  | |  | |  | |  | |  | | |  | |
| Hollis (2015) |  | |  | |  | |  | |  | |  | |  | |  | |  | | |  | |
| Hopkins (2023) |  | |  | |  | |  | |  | |  | |  | |  | |  | | |  | |
| Hussain (2018) |  | |  | |  | |  | |  | |  | |  | |  | |  | | |  | |
| Karamian (2022) |  | |  | |  | |  | |  | |  | |  | |  | |  | | |  | |
| Maggio (2015)ᵃ |  | |  | |  | |  | |  | |  | |  | |  | |  | | |  | |
| Moine (2016) |  | |  | |  | |  | |  | |  | |  | |  | |  | | |  | |
| Morris (2020) |  | |  | |  | |  | |  | |  | |  | |  | |  | | |  | |
| Okoro (2023) |  | |  | |  | |  | |  | |  | |  | |  | |  | | |  | |
| Peppard (2017) |  | |  | |  | |  | |  | |  | |  | |  | |  | | |  | |
| Perez (2024) |  | |  | |  | |  | |  | |  | |  | |  | |  | | |  | |
| Rodriquez de Castro (2020) |  | |  | |  | |  | |  | |  | |  | |  | |  | | |  | |
| Salm (2020) |  | |  | |  | |  | |  | |  | |  | |  | |  | | |  | |
| Scheck (2017) |  | |  | |  | |  | |  | |  | |  | |  | |  | | |  |  |
| Sommerstein (2015) |  | |  | |  | |  | |  | |  | |  | |  | |  | | |  |  |
| Stitely (2023)ᵃ |  | |  | |  | |  | |  | |  | |  | |  | |  | | |  |  |
| Unger (2014) |  | |  | |  | |  | |  | |  | |  | |  | |  | | |  |  |
| Wu (2016) |  | |  | |  | |  | |  | |  | |  | |  | |  | | |  |  |
| Young (2015)ᵃ |  | |  | |  | |  | |  | |  | |  | |  | |  | | |  |  |
|  | |  | |  | |  | |  | |  | |  | |  | |  | |  | | |  |
|  |  | |  | | **Low** | |  | |  | |  | |  | |  | |  | | |  |  |
|  |  | |  | | **Some concerns** | |  | |  | |  | |  | |  | |  | | |  |  |
|  |  | |  | | **Serious** | |  | |  | |  | |  | |  | |  | | |  |  |

ᵃ RCTs assessed with Revised Cochrane risk-of-bias tool for randomized trials (RoB 2)

**Table S4.** GRADE assessment

Meta-analysis of RCTs:

The Grading of Recommendation, Assessment, Development and Evaluation (GRADE) approach was used to grade certainty of evidence using a minimally contextualized approach on the following five domains: risk of bias, inconsistency, indirectness, imprecision and publication bias.^1^ The minimally important difference was not calculated as the SSI incidence was 0 in data of present meta-analysis of included RCTs for patients receiving standard SAP.^2^

Since all included studies are randomized controlled trials, the starting certainty of evidence was high. Downgrading can be necessary due to the following reasons:

- Risk of bias

Since risk of bias assessment resulted in ‘low’ or ‘some concerns’ among included studies, downgrading was not necessary.

- Inconsistency

For inconsistency no downgrading was necessary (*I^2^* = 0%, τ^2^ = 0).

- Indirectness

The included RCTs involved only obstetric surgery with female patients, rather than a broader surgical population with both male and female patients. One level of downgrading was needed as pathogen and tissue penetrations are different across male and female patients. Other than that, the body of evidence of the studies does represent the PICO elements of interest.^3^

- Imprecision

There were very few events and the CI overlapped the thresholds of interest. Therefore, we downgraded two levels for imprecision.

- Publication bias

Rating down one level for publication bias was necessary because the evidence consists of a small number of small studies.

|  | **Certainty assessment** | | | | | | | **No of patients** | | **Effect** | | **Certainty** | |
| --- | --- | --- | --- | --- | --- | --- | --- | --- | --- | --- | --- | --- | --- |
|  | No of studies | Study design | Risk of bias | Inconsistency | Indirectness | Imprecision | Publication bias | Weight-based dosing | Standard dosing | Risk Difference  (95% CI) |  | |  |
| SSI | 3 | RCT | Not serious | Not serious | Serious  (-1 downgrade) | Serious  (-2 downgrade) | Serious  (-1 downgrade) | 1/51 (2.0%) | 0/52 (0%) | 2.02 (-3.15 to 7.19) |  | | ⨁◯◯◯ very low |
| CI = confidence interval; GRADE = Grading of Recommendations, Assessment, Development and Evaluation; PICO = population, intervention, comparison and outcomes; SSI = surgical site infection | | | | | | | | | | | | | |

Meta-analysis of observational studies

The Grading of Recommendation, Assessment, Development and Evaluation (GRADE) approach was used to grade certainty of evidence using a minimally contextualized approach on the following five domains: risk of bias, inconsistency, indirectness, imprecision and publication bias.^1^ The minimally important difference was defined as 0.92% based on the default for appreciable benefit and harm of 25% and the SSI incidence of 3.7% in data of present meta-analysis of included observational studies for patients with standard SAP.^2^ We evaluated imprecision taking the minimally important differences into account.

Since all included studies are observational cohort studies, the starting certainty of evidence was low. Downgrading can be necessary due to the following reasons:

- Risk of bias

All six included studies showed high risk of bias. Downgrading for the risk of bias was thereby necessary. Since all included studies showed high risk of bias, no sensitivity analysis was performed.

- Inconsistency

For inconsistency no downgrading was necessary (*I^2^* = 0%, τ^2^ = <0.0001).

- Indirectness

All but one study included only orthopaedic patients rather than a broader surgical population. A sensitivity analysis excluding the study using metronidazole next to cefazolin was performed. The results were comparable to the primary analysis (RD -1.99%; 95% CI: -3.12 to -0.86 and 95% PI -3.96 to -0.01). The tissue penetrations are expected to be comparable, so no downgrade was needed. Other than that, the body of evidence of the studies does represent the PICO elements of interest.^3^

- Imprecision

The CI did not overlap the thresholds of interest, so no downgrading was needed.

- Publication bias

Rating down one level for publication bias was necessary because the evidence consists of a small number of studies.

|  | **Certainty assessment** | | | | | | | **No of patients** | | **Effect** | | **Certainty** | |
| --- | --- | --- | --- | --- | --- | --- | --- | --- | --- | --- | --- | --- | --- |
|  | No of studies | Study design | Risk of bias | Inconsistency | Indirectness | Imprecision | Publication bias | Weight-based dosing | No weight-based dosing | Risk Difference  (95% CI) |  | |  |
| SSI | 6 | Observational | Serious  (-1 downgrade) | Not Serious | Not serious | Not serious | Serious  (-1 downgrade) | 470/41,733 (1.1%) | 140/3,821  (3.7%) | -1.93 (-2.84 to  -1.02) |  | | ⨁◯◯◯ very low |
| CI = confidence interval; GRADE = Grading of Recommendations, Assessment, Development and Evaluation; PICO = population, intervention, comparison and outcomes; SSI = surgical site infection | | | | | | | | | | | | | |

**References**

1. Schünemann HB JG GO, A. GRADE Handbook for grading quality of evidence and strength of recommendations. Updated October 2013. 2013 [Available from: <https://gdt.gradepro.org/app/handbook/handbook.html>.

2. Zeng L B-PR, Hultcrantz M, Siemieniuk RAC, Santesso N, Traversy G, et al. GRADE guidelines 32: GRADE offers guidance on choosing targets of GRADE certainty of evidence ratings. 2021Doi.<https://doi.org/10.1016/j.jclinepi.2021.03.026>

3. Zhang Y A-CP, Guyatt GH, Yepes-Nuñez JJ, Akl EA, Hazlewood G, et al. GRADE Guidelines: 19. Assessing the certainty of evidence in the importance of outcomes or values and preferences-Risk of bias and indirectness. J Clin Epidemiol 111:94-1042019Doi. <https://doi.org/10.1016/j.jclinepi.2018.01.013>

**Figure S1.** Sensitivity analysis using an arcsine difference model.

**
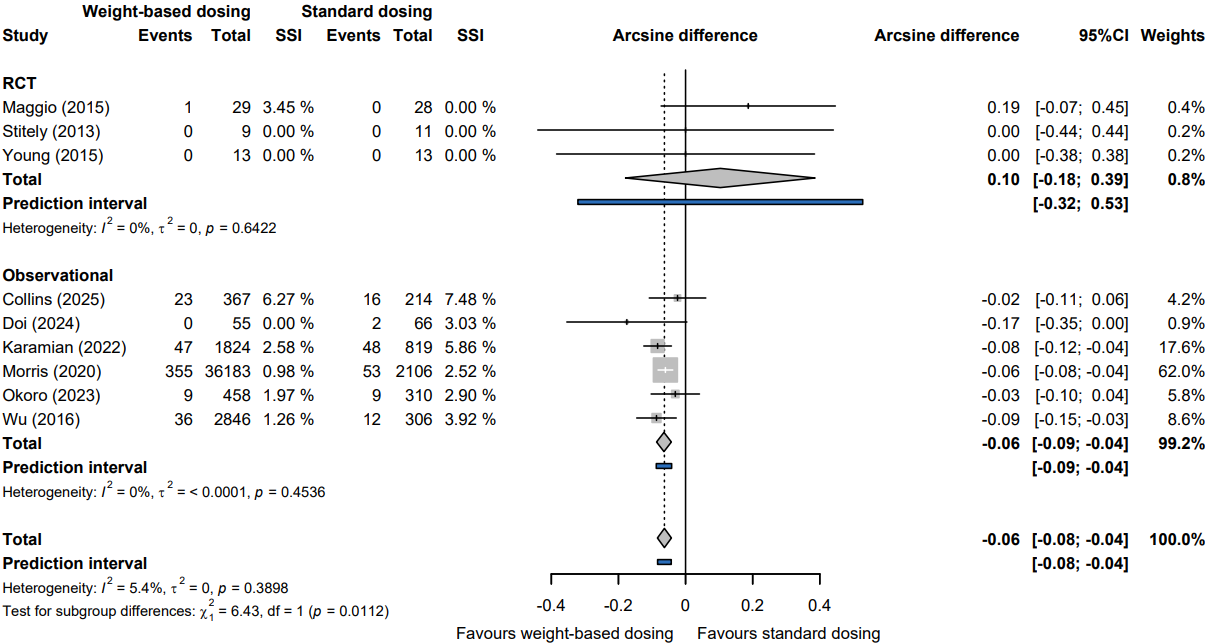
**

Prediction intervals, represented by the horizontal blue bars, illustrate the expected range of true effects.

**Figure S2.** Sensitivity analysis of studies using weight-based dosing of cefazolin only in elective surgery procedures.

**
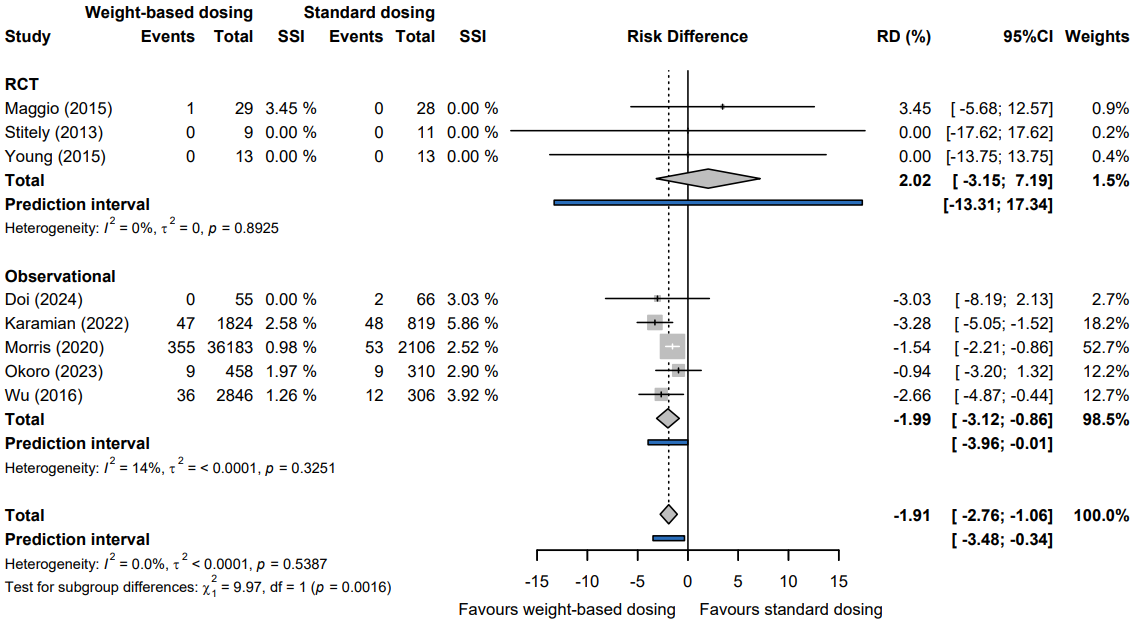
**

Prediction intervals, represented by the horizontal blue bars, illustrate the expected range of true effects.

**Figure S3.** Meta-analysis of RCTs and observational studies using weight-based dosing of SAP versus standard dosing in elective surgery procedures, using the OR.


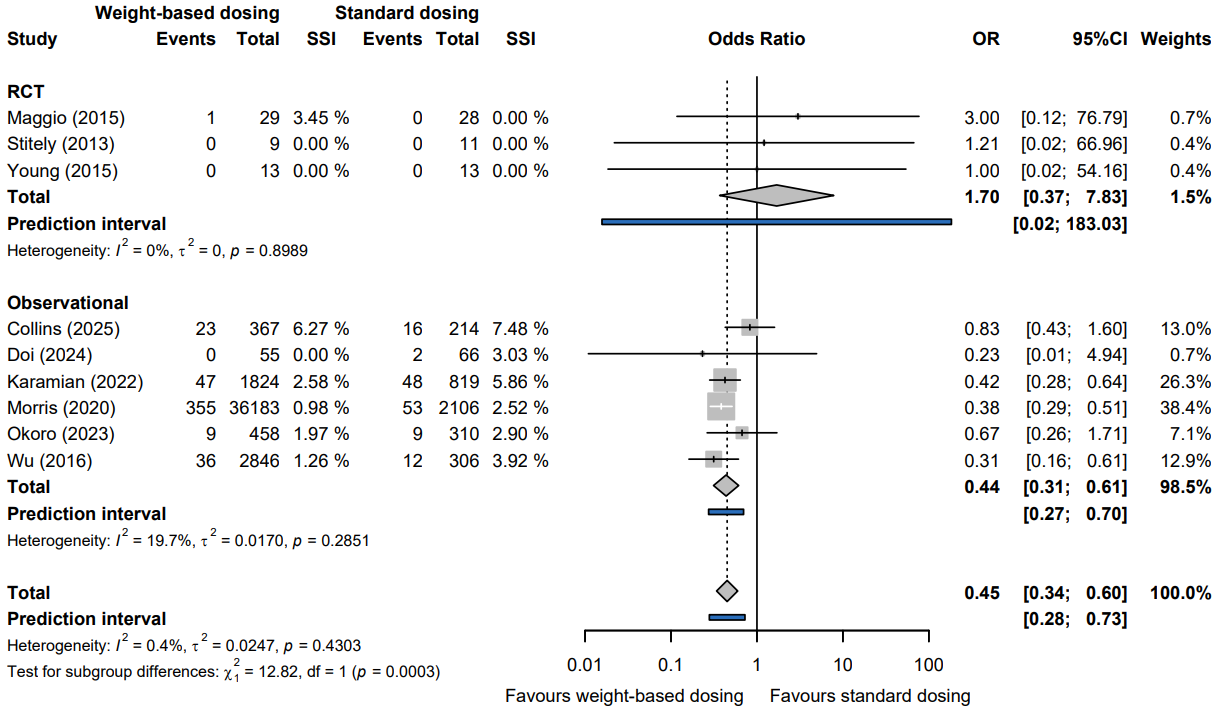


Prediction intervals, represented by the horizontal blue bars, illustrate the expected range of true effects.

**Figure S4.** Overview of SSI rates in each individual study arm focusing on patients with overweight and obesity using cefazolin.

**
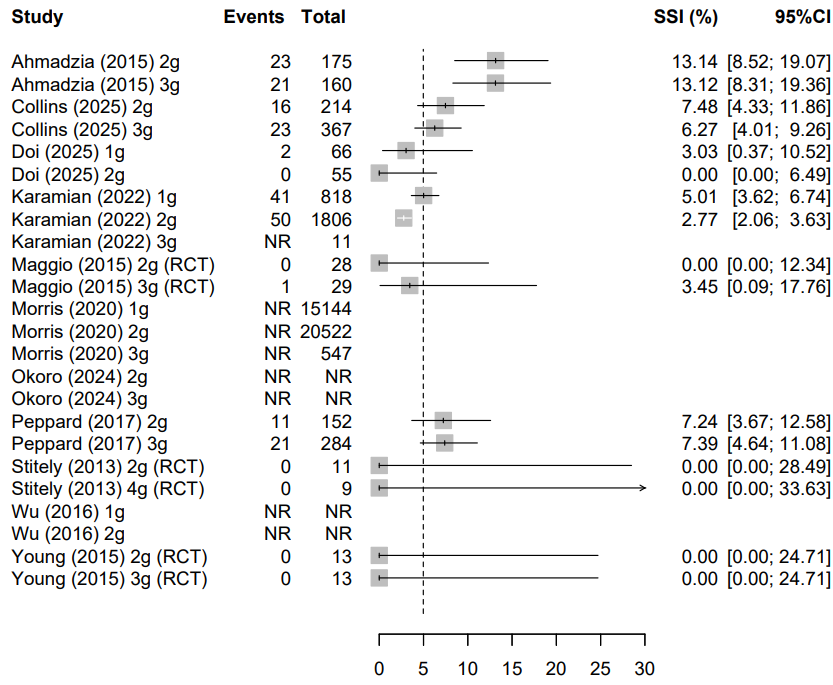
**

NR Not reported

**Figure S5.** Overview of SSI rates in each individual study arm focusing on patients with overweight and obesity using different antimicrobial agents

5a) Studies using cefotetan + cefoxitin

**Study**

Banoub (2018) 2g

Banoub (2018) 3g

**Events**

29

8

**Total**

140

35

0

5

10

15

20

25

30

**SSI (%)**

20.71

22.86

**95%CI**

[14.33; 28.38]

[10.42; 40.14]

5b) Studies using cefuroxime

**Study**

Hasler (2021) 1.5g

Hasler (2021) 3g

Sommerstein (2021) 1,5g

Sommerstein (2021) 3g

**Events**

16

8

747

462

**Total**

4010

3096

24394

13246

0

5

10

15

20

25

30

**SSI (%)**

0.40

0.26

3.06

3.49

**95%CI**

[0.23; 0.65]

[0.11; 0.51]

[2.85; 3.29]

[3.18; 3.81]

5c) Studies using cefuroxime + metronidazole

**Study**

Salm (2021) 1.5/0.5g

Salm (2021) 3/1g

**Events**

95

73

**Total**

546

1615

0

5

10

15

20

25

30

**SSI (%)**

17.40

4.52

**95%CI**

[14.31; 20.84]

[ 3.56; 5.65]

5d) Studies using vancomycin

**Study**

Catanzano (2014) <1g

Catanzano (2014) 1g

**Events**

6

0

**Total**

149

45

0

5

10

15

20

25

30

**SSI (%)**

4.03

0.00

**95%CI**

[1.49; 8.56]

[0.00; 7.87]

5e) Studies using cefazolin + metronidazole


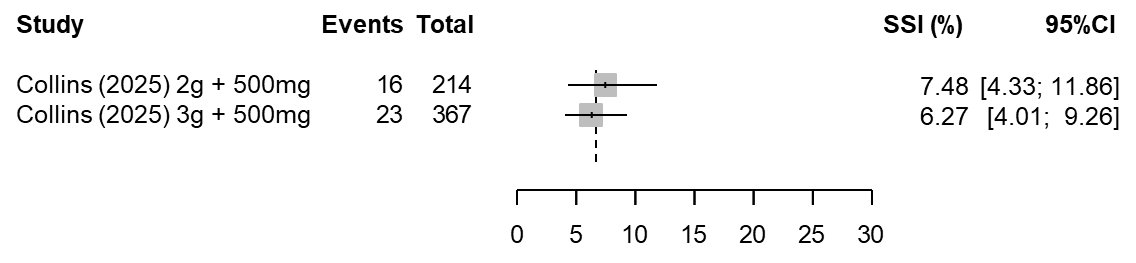

Supplement: zrag015_Supplementary_Data [file zrag015_supplementary_data.docx]
